# The impact of immune dysfunction on perioperative complications in surgical COVID-19 patients: an imperative for early immunonutrition

**DOI:** 10.1186/s13037-022-00323-y

**Published:** 2022-04-01

**Authors:** Vincent P. Stahel, Samson D. Blum, Pratibha Anand

**Affiliations:** 1 University of Colorado (CU), Boulder, CO 80309 USA; 2 University of Colorado, School of Medicine, Aurora, CO 80045 USA

**Keywords:** Coronavirus, COVID-19, Surgical complications, Immune dysfunction, Immunonutrition

## Abstract

Surgical patients with coronavirus disease 2019 (COVID-19) are vulnerable to increased perioperative complications and postoperative mortality, independent of the risk for contracting COVID-19 pneumonia after endotracheal intubation for general anesthesia. The presumed root cause of postoperative infections, microvascular soft tissue injuries and thromboembolic complications is largely attributed to the profound immune dysfunction induced by COVID-19 as a result of complement activation and the “cytokine storm”. The empirical therapy with anti-inflammatory agents has been shown to attenuate some of the adverse effects of systemic hyperinflammation in COVID-19 patients. In addition, the proactive concept of “immunonutrition” may represent a new promising avenue for mitigating the complex immune dysregulation in COVID-19 and thereby reduce the rates of surgical complications and postoperative mortality. This letter provides a narrative summary of the current state-of-the-art in the field of immunonutrition as it pertains to surgical patient safety in COVID-19 patients.

## Background

Postoperative mortality rates of surgical patients with coronavirus disease 2019 (COVID-19) have been shown to be dramatically increased compared to COVID-19 negative patients who undergo surgical procedures [1–4]. The increased postoperative mortality rate in COVID-19 patients has been observed in symptomatic as well as asymptomatic patients and applies to both elective and urgent/emergent surgical procedures [5–12]. Current medical treatment options for COVID-19 are limited, prompting the necessity to explore alternative treatment strategies that are both effective and widely available [13–16]. The emerging field of immunonutrition provides a novel potential mechanism to decrease perioperative morbidity in COVID-19 patients and subsequently decrease the risk of surgical complications [17, 18].

**Fig. 1:**
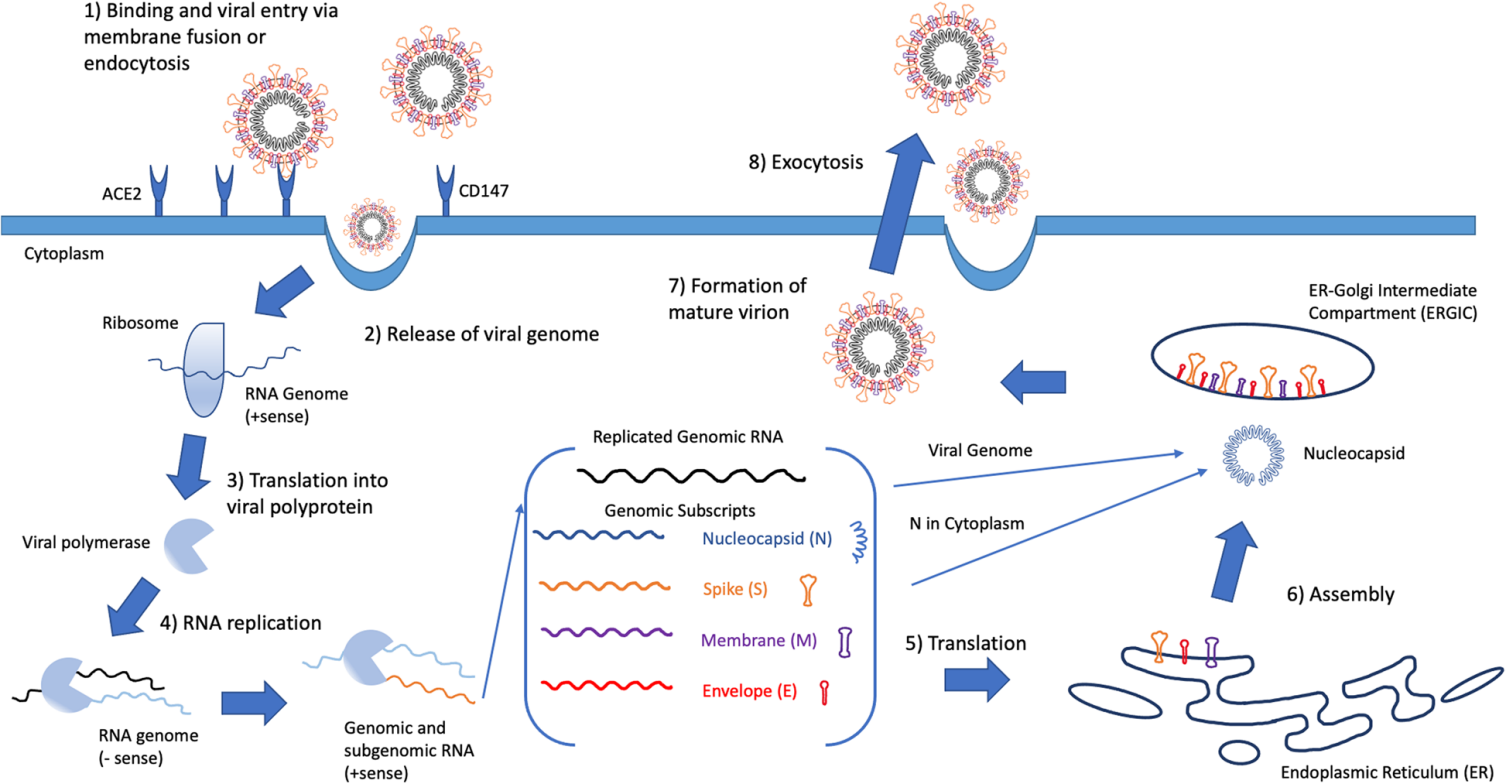
Schematic depiction of the SARS-CoV-2 cellular infection mechanisms. Adopted and modified from: Lebeau G et al., Deciphering SARS-CoV-2 virologic and immunologic features. *Int. J. Mol. Sci.* 2020, 21(16):5932 [19]

The mechanism of cellular infection by SARS-CoV-2 is depicted schematically in Fig. 1. The complex immune dysfunction in patients with COVID-19 has been implicated in adverse outcomes due to uncontrolled hyperinflammation, hypercoagulability associated with thromboembolic complications, and ultimately to delayed organ failure and death [20–22]. The “cytokine storm” (or “cytokine release syndrome”) and the activation of the complement cascade have been identified as key mechanisms contributing to the immunopathology of COVID-19 [23–25]. A wide spectrum of empirical treatment modalities have been widely applied during the pandemic in off-label indications to attenuate the hyperinflammatory response to coronavirus infection. These include antirheumatic agents, cytokine inhibitors, corticosteroids, intravenous immunoglobulin, complement inhibitors, and other novel anti-inflammatory molecules [26–29]. However, there is a lack of effective and specific therapeutics that may help reduce the risk of immune dysfunction-associated surgical complications in COVID-19 patients [15]. The benefit of immunonutrition in attenuating hyperinflammation and adverse outcomes in the setting of major surgery or major injuries has been established for many decades in the pertinent literature [30–32].

## Immunonutrition in COVID-19

Immunonutrition is an evolving concept designed to improve the potential of modulating the immune system in at-risk patients by providing high-dose supportive nutrients during a phase of increased vulnerability, e.g. due to infection, trauma, or surgery [30–34]. Micronutrients, such as vitamin C, D3, zinc, and selenium have been shown to play important supportive roles in antioxidant, anti-inflammatory, antithrombotic, antiviral, and immuno-modulatory functions in COVID-19 patients [33–37]. The present letter was designed to provide a pragmatic rationale to consider immunonutrition as an adjunct in the perioperative management of the vulnerable cohort of COVID-19 patients who require surgical interventions.

### Vitamin C

Ascorbic acid, commonly known as vitamin C, plays a vital role in regulating immune function and has been shown to support the innate and the adaptive immune system [38]. Vitamin C promotes neutrophil activation, recruitment, and phagocytosis, thereby contributing to anti-viral immune mechanisms [39, 40]. Importantly, Vitamin C also serves to prevent tissue damage by decreasing neutrophil necrosis and promoting subsequent clearance by macrophages and works on the skin level by promoting the epithelial barrier, enhancing protection against environmental strain [41]. Current data has highlighted the importance of Vitamin C in combating infection and has shown that Vitamin C deficiency results in weakened immune function resulting in higher rates of infection [41]. Intravenous (IV) supplementation of Vitamin C in patients with COVID-19 has resulted in a marked decrease of inflammatory markers including D-Dimer and ferritin and highlighted potential benefits including shortened recovery times, decreased length of mechanical ventilation, and time spent in the ICU [6]. The use of IV vitamin C supplementation in COVID-19 patients continues to be of limited study, however early anecdotal evidence supports its beneficial mechanisms by reducing overall mortality rates in COVID-19 patients including those critically ill [39, 42, 43].

### Vitamin D3

Vitamin D3 (cholecalciferol) is historically known for its roles in calcium homeostasis and bone health, and also influences the innate and adaptive immune system by supporting antiviral mechanisms and attenuating inflammation [44, 45]. Decreased serum levels of vitamin D3 have been shown to significantly correlate with increased morbidity and mortality in COVID-19 patients [18, 46–48]. From a therapeutic immunonutrition perspective, the supplementation with vitamin D3 has been demonstrated to improve outcomes in COVID-19 patients and to decrease ICU admissions and mortality [49].

### Zinc

The discovery of the interaction between zinc and immune function dates back to the 1960’s [50]. Although the exact functions of zinc in the immune system remain to be further elaborated, it has shown to exhibit anti-inflammatory and antioxidant capacities and serves roles in immune cell maturation and differentiation [48, 51]. In COVID-19, the administration of zinc in high doses has been shown to suport the immune system’s antiviral activity and to inhibit coronavirus replication [41, 52, 53].

### Glutamine

Glutamine is considered an essential amino acid in cases of catabolic disease and is the most abundant amino acid in the body and primarily found in skeletal muscle and the lungs [54, 55]. Glutamine serves as the fuel for various immune and intestinal cells including lymphocytes and macrophages, as well as aiding in the creation of proteins, glucose, and other amino acids [55]. These functions allow Glutamine to mediate immunological function and support antioxidant effects [56]. Other important functions of Glutamine include supporting gut mucosa, muscle growth, and nitrogen transport between organs [56]. L-glutamine has been shown to have beneficial impacts on COVID-19 patients by inhibiting inflammatory processes resulting in decreased length of hospitalization and lower rates of ICU admissions [57]. Based on the demonstrated disruption of the metabolism of amino acids, including glutamine, in COVID-19 patients, in conjunction with the proven immunomodulatory functions of glutamine, this amino acid may present an ideal nutrient as an adjunct for surgical patients with COVID-19 infections [32, 58].

### Omega-3 fatty acids

Omega-3 fatty acids are polyunsaturated fatty acids consisting of both eicosapentaenoic (EPH) and docosahexaenoic (DHA) acids [59]. Polyunsaturated fatty acids have long been known for antiviral properties and are associated with the gut microbiota and gut-brain axis [60]. Recent studies have shown that high levels of polyunsaturated fatty acids (omega-3 or omega-6) decrease the susceptibility to coronavirus infections and are protective against developing severe COVID-19 disease [61–64]. A double-blind randomized clinical trial on critically ill patients with COVID-19 demonstrated that the nutritional omega-3 supplementation improved clinical outcomes including renal and respiratory function [65]. However, the high-dose supplementary use of omega-3 fatty acids has also been cautioned, due to EPA and DHA causing cell membranes to become more susceptible to oxidative stress and related cellular toxicity [59].

## Conclusion

In light of the limited specific treatment options for surgical COVID-19 patients who are at risk of high postoperative complication rates and adverse outcomes, supportive immunonutrition appears to represent a safe, feasible, cost-effective, and pragmatically intuitive concept to mitigate the perioperative inflammation and adverse effects related to immune dysfunction [30, 35]. The micronutrients described in this review (vitamin C, D3, zinc, glutamine, and omega-3 fatty acids) have all shown to have a positive influence on the immune system and boost immune function through various pathways and mechanisms. The recommended dosing ranges of these immunonutrients for COVID-19 patients are provided in Table 1. Preliminary insights from case studies and randomized trials support the notion that immunonutrition decreases the severity of disease and risk of complications in COVID-19 patients by attenuating the inflammatory response and supporting antiviral activity by the immune system [30, 34, 35].

**Table 1:** Recommended dosing ranges for immunonutrients in COVID-19

Pharmaconutrient	Immune mechanisms	Recommended intake for adults	Recommended higher dose in COVID-19 (limited to short-term treatment)	Potential side effects	Dietary sources
Vitamin C	-Neutrophil allocation -Phagocytosis -Macrophage clearance	65–90 mg per day	2000 mg per day	Gastrointestinal	Red peppers, oranges, kiwi, strawberries, and tomato juice
Vitamin D3	-Antiviral and anti-inflammatory properties	15–20 mcg per day	1000 units per day	Gastrointestinal; urolithiasis; muscle weakness	Cod, trout, salmon, white mushrooms, and milk
Zinc	-Immune cell maturation -Intercellular communication	8–11 mg per day	200 mg per day	Gastrointestinal; headaches	Beef, pork, poultry, oysters, crab, lobster, and breakfast cereals
L-Glutamine (Amino Acid)	-Fuel for immune cells -Supports glucose, protein and amino acid production	0.8 g/kg body weight per day	30 mg per day	Gastrointestinal; dizziness	Beef, pork, poultry, dairy products, fish, and beans
Ω-3 Fatty Acids	-Anti-inflammatory properties -Supports gut microbiota	1.1–1.6 mg per day	1000 mg per day	Gastrointestinal; headaches	Flaxseed, soybean, and canola oils, salmon, herring, and mackerel.

## Data Availability

Please contact the authors for data requests.
